# *Characterization* of a Novel Family of Contilisant + Belinostat Multitarget Small Molecules in Glioblastoma

**DOI:** 10.3390/ph19010020

**Published:** 2025-12-22

**Authors:** Aizpea Artetxe-Zurutuza, Nerea Iturrioz-Rodriguez, Joseba Elizazu, Raul Garcia-Garcia de Garayo, Irati de Goñi, Jhonatan Vergara, Mireia Toledano-Pinedo, Alicia Porro-Pérez, Mikel Azkargorta, Felix Elortza, Jose Luis Marco-Contelles, Nicolás Sampron, Ander Matheu

**Affiliations:** 1 Cellular Oncology Group, Biogipuzkoa (Biodonostia) Health Research Institute, 20014 San Sebastian, Spainnerea.iturriozrodriguez@bio-gipuzkoa.eus (N.I.-R.); jhonatanandres.vergaraarce@bio-gipuzkoa.eus (J.V.);; 2 Neurosurgery Service, Donostia University Hospital, 20014 San Sebastian, Spain; 3 Laboratory of Medicinal Chemistry, Institute of General Organic Chemistry (CSIC), 28006 Madrid, Spainaliporro@ucm.es (A.P.-P.); jlmarco@iqog.csic.es (J.L.M.-C.); 4 Proteomics Platform, CIC bioGUNE, Basque Research and Technology Alliance (BRTA), CIBERehd, 48160 Derio, Spain; 5 Centre for Biomedical Network Research on Rare Diseases (CIBERER), ISCIII, 28029 Madrid, Spain; 6 Centre for Biomedical Network Research on Frailty and Healthy Aging (CIBERFES), ISCIII, 28029 Madrid, Spain; 7 IKERBASQUE, Basque Foundation for Science, 48009 Bilbao, Spain

**Keywords:** glioblastoma, MSM, HDAC inhibition, glioma stem cells, proliferation

## Abstract

**Background**: Glioblastoma is the most common and malignant primary brain tumor in adults, with current treatment presenting limited effectiveness. Therapeutic resistance stems largely from its marked molecular and cellular heterogeneity. Multitarget small molecules (MSMs) have emerged as a promising strategy for treating complex diseases such as cancer. In the present work, we generated a novel family of indole-based MSMs engineered to inhibit histone deacetylases (HDACs), monoamine oxidases (MAOs) and cholinesterases (ChEs) while simultaneously acting as histamine H_3_ receptor (H3R) antagonists and sigma-1 receptor (S1R) agonists. **Methods**: To accomplish this, we combined selected pharmacophoric moieties from the parent compounds Contilisant and the HDAC pan-inhibitor Belinostat. Nine MSMs were synthesized. **Results**: Most of them showed cytotoxic activity in glioma cells. Among them, three molecules (MTP142, MTP156 and MTP150) were prioritized based on potency; these compounds impaired glioma stem cell (GSC) activity and were predicted to cross the blood–brain barrier. In vivo and multi-omic analyses centered on MTP150 showed significant tumor growth inhibition, both as monotherapy and in combination with temozolomide (TMZ). Transcriptomic and proteomic profiling of patient-derived GSCs revealed MTP150-induced disruption of cell cycle regulation pathways. **Conclusions**: Our data reveal the efficacy of a novel family of MSMs in the pre-clinical setting of glioblastoma.

## 1. Introduction

Cancer constitutes one of the main health and economic issues of the 21st century, with an incidence of approximately 20 million diagnoses and 10 million deaths annually, accounting for nearly 17% of global mortality [1]. Although brain tumors do not represent a cancer type with a high incidence rate, they have a great impact in terms of morbidity and mortality [2]. Glioblastoma (GB), also known as IDH-wildtype WHO grade 4 glioma, is the most common and malignant primary brain tumor. It has a very poor prognosis, with an overall survival of around 15 months and a 5-year survival rate of below 5% [2]. Currently, treatment of GB is based on the maximal surgical resection of the tumor, followed by radiotherapy and chemotherapy with TMZ [3]. However, GB is characterized by high intratumoral and intertumoral heterogeneity, which poses a major challenge for treatment effectiveness [4]. Moreover, the presence of a subpopulation of glioma stem cells (GSCs), which are quiescent and pluripotent cells with the capacity of self-renewal, further complicates treatment of the disease, as this subpopulation of cells often leads to therapy resistance and recurrences [5]. Altogether, this heterogeneity reflects the need for the development of novel effective treatments.

Over recent decades, drug design research has largely focused on single-target compounds designed to specifically bind to a unique biological entity with high selectivity, minimizing unwanted off-target effects [6]. However, nowadays, it is clear that many diseases, including cancer and neurodegenerative disorders, arise from disruptions across multiple signaling pathways. Consequently, single-target treatments often fail to achieve meaningful clinical benefit in complex diseases [7,8]. In contrast to the “one-molecule, one-target” paradigm, multitarget small molecules (MSMs) have emerged as a powerful strategy for treating multifactorial diseases [9,10]. In fact, the aforementioned diseases often share overlapping pathogenic mechanisms, making them suitable for drug repurposing approaches [10]. MSMs can also reduce adverse effects while enhancing therapeutic efficacy [11], and offer additional advantages such as more predictable pharmacokinetics and pharmacodynamics [12]. Their multitarget activity may produce synergistic effects even when their individual target potencies are modest [13]. Additionally, the use of a unique multitarget compound can improve patient adherence [14].

Indole is a heterocycle scaffold commonly found in natural products. Its ability to donate or accept hydrogen bonds enables interactions with diverse molecular targets, resulting in broad biological activity. Because of this versatility, indole-based compounds have become valuable frameworks for therapeutic development [15]. These molecules modulate processes involved in cancer, including cell cycle and proliferation, DNA repair, and cell death. Notably, some indole-based drugs have already been approved by the FDA [16].

Contilisant, developed through a multitarget design strategy incorporating an indole scaffold, is a tetratarget small molecule able to inhibit monoamine oxidases (MAOs) and cholinesterases (ChEs) while functioning as a histamine H_3_ receptor (H3R) antagonist and sigma 1 receptor (S1R) agonist. Originally generated for the treatment of neurodegenerative diseases, Contilisant has demonstrated neuroprotective, antioxidant, permeable, and non-toxic properties, and alleviates Aβ_1–42_-induced cognitive deficits in vivo [17].

HDACs regulate multiple biological processes, including transcription, metabolism, angiogenesis, oxidative stress, DNA repair, cell cycle progression and apoptosis [18]. Aberrant HDAC expression has been detected in both solid and hematological malignancies, including GBM, where elevated levels correlate with advanced stages of the disease and poor prognosis [19]. Functional studies have shown that HDACs promote proliferation, invasion, migration, angiogenesis, and resistance to apoptosis [20], and regulate the activity of GSCs [21]. In addition, overexpression of these enzymes has also been linked to the development of TMZ resistance [22]. These findings have led to the development of HDAC inhibitors as promising agents for GBM therapy. The FDA has approved some pan-HDAC inhibitors, including SAHA and Belinostat for hematological cancers, and several clinical trials have explored their activity in solid tumors including glioblastoma [23].

The inhibition of histone deacetylases (HDACs) offers a promising alternative that warrants further investigation for multifactorial diseases. Beyond HDACs, other enzymes and receptors targeted by MSMs are also dysregulated in GBM. MAO expression is elevated in multiple cancers, including glioblastoma [24], and MAO inhibitors reduce glioma viability in vitro and tumor growth in vivo [25]. ChEs are likewise upregulated in gliomas and correlate with tumor progression [26,27], while activation of acetylcholine (ACh) receptors can suppress proliferation and induce apoptosis [28]. Expression of H_3_R and sigma receptors (S1R and S2R) is increased in glioblastomas. Consequently, several multitarget compounds against HDACs and additional Contilisant targets have been developed. In fact, a dual MAO-A and HDAC inhibitor has been shown to inhibit cell growth and induce cell death in glioma, as well as to prolong survival [29]. In addition, a S1R agonist with HDAC inhibitory capacity has also shown antitumor effects in GB, inducing cell death in both TMZ-sensitive and TMZ-resistant cells, and prolonging survival in in vivo orthotopic mouse models [30].

As a consequence, we developed a new family of compounds combining Contilisant with the pan-HDAC inhibitor Belinostat [31]. In particular, Belinostat is an HDAC inhibitor acting on class I (HDACs 1, 2, 3, and 8), and II (HDACs 4, 5, 6, 7, 9 and 10) enzymes, and is FDA-approved for the treatment of relapsed or refractory peripheral T-cell lymphoma, with a favorable safety profile and toleration [32]. In addition, Belinostat has been investigated as cytostatic agent in solid malignancies, including glioblastoma, where it induces apoptosis and inhibits cell growth in a dose-dependent manner [33]. A pilot clinical trial revealed synergistic effects when Belinostat was combined with radiotherapy and TMZ chemotherapy [34]. Furthermore, certain Belinostat-derived MSMs have shown cognitive benefits with no apparent toxicities [35]. In this study, we characterized their effect against cancer.

## 2. Results

### 2.1. Novel Family of MSMs Is Cytotoxic for Glioma Cells

**Figure 1:**
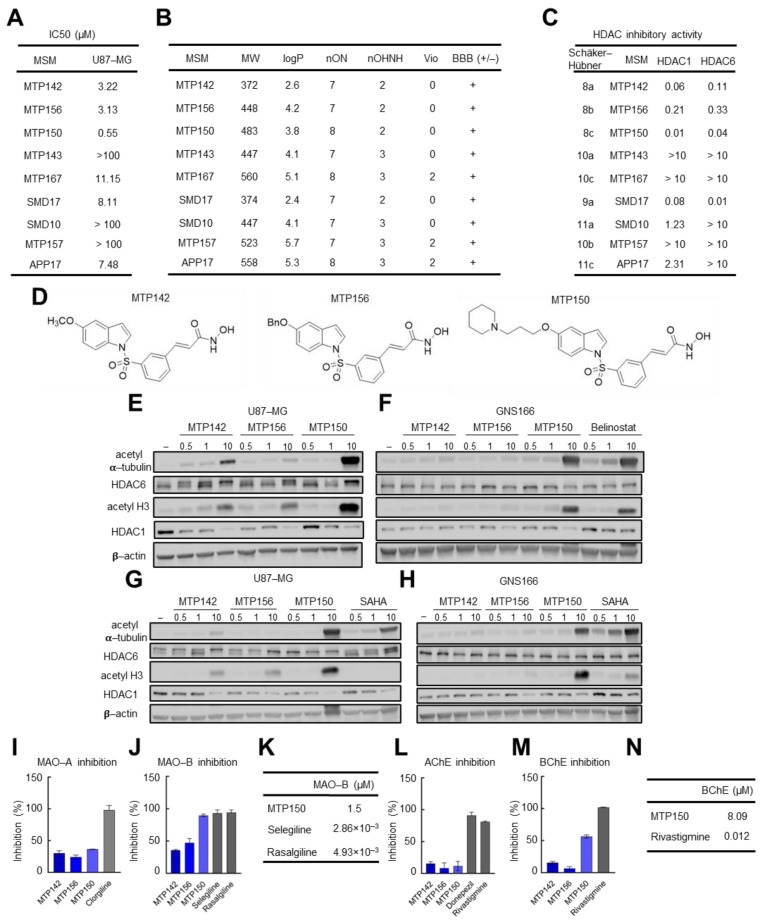
**Novel MSMs are cytotoxic for GB and inhibit target genes.** (**A**) IC_50_ values of the compounds of the novel family of MSMs in the U87 cell line. (**B**) Table summarizing the physicochemical properties of the four MSMs and their predicted ability to cross the BBB. (**C**) IC_50_ values of novel MSMs for HDAC1 and HDAC6. (**D**) Chemical structure of the three MTP molecules. (**E**–**H**) Representative Western blot of HDAC6 and HDAC1 and their targets acetylated α-tubulin and acetylated H3 in (**E**–**G**) U87-MG and (**F**–**H**) GNS166 cells treated with increasing concentrations of MTP142, MTP156, MTP150 and Belinostat or SAHA. (**I**–**K**) MAO-A and MAO-B inhibitory capacity of MTP142, MTP156 and MTP150. (**L**,**M**) AChE and BChE inhibitory capacity of MTP142, MTP156 and MTP150, (**N**) BChE IC50 values of MTP150 and the reference compound Rivastigmine.

We first tested the cytotoxic effect of nine novel MSMs synthesized by combining Contilisant and Belinostat. Nearly all molecules displayed cytotoxicity in U87-MG cells, with IC_50_ values ranging between 0.5 and 11 µM (Figure 1A). Among them, MTP142, MTP156 and MTP150 presented the highest cytotoxicity, with IC_50_ values of 3.22, 3.13 and 0.55 µM, respectively. Based on their molecular structures and online prediction tools, they were predicted to cross the BBB, a major bottleneck in glioblastoma drug development. In addition, most MSMs, including the three most potent compounds (MTP142, MTP156 and MTP150) presented no violations of Lipinski’s “rule of 5”, which states that “drug-like” molecules typically present logP ≤ 5, molecular weight ≤ 500 g/mol, ≤10 hydrogen bond acceptors, and ≤5 hydrogen bond donors (Figure 1B). Thus, compounds with fewer violations of this rule exhibit more favorable drug-like properties [36]. Collectively, these results indicate that the novel MSMs have strong drug-like profiles. They also showed an absence of predicted toxicity risks, including mutagenic, tumorigenic, and irritant characteristics.

Next, we evaluated the MSMs’ effects on their molecular targets. HDAC inhibitory activity, measured by IC_50_ for HDAC1 and HDAC6, was the highest in MTP150, MTP142, SMD17 and MTP156. Notably, all four compounds displayed IC_50_ values below 1 µM for both enzymes, indicating that they function as effective pan-HDAC inhibitors (Figure 1C) [31]. Consistently, MSMs showed stronger inhibitory activity against HDAC1 than HDAC6. In detail, MTP142, MTP156, MTP150 and SMD17 exhibited the most potent inhibition, with IC_50_ values below 0.3 µM for both HDACs. Among them, MTP150 demonstrated the greatest potency, with IC_50_ values of 0.01 and 0.04 µM for HDAC1 and HDAC6, respectively (Figure 1C). Therefore, MTP142, MTP150 and MTP156 MSMs (their chemical structure is presented in Figure 1D) were selected for further characterization.

Next, the effect on the protein levels of HDAC6 and HDAC1, as well as their main targets, acetylated α-tubulin and acetylated H3, was studied by Western blot. Interestingly, increased levels of both acetylated α-tubulin and acetylated H3 proteins were observed in response to increasing concentrations (0.5, 1 and 10 µM) of the three MSMs in U87-MG cells (Figure 1E). In agreement with the IC_50_ values, MTP150 was the molecule showing the highest levels of acetylated proteins, thereby revealing the strongest HDAC inhibition. Additionally, the effect of the MSMs was assessed in patient-derived GNS166 and compared with that of the HDAC pan-inhibitors Belinostat and SAHA. MSMs also promoted increased expression of acetylated proteins, although primarily at the highest concentrations in GNS166 cells (Figure 1F). Notably, MTP150 induced higher levels of acetylated α-tubulin and H3 than both Belinostat and SAHA in both cell types (Figure 1F–H). Finally, the effects of MSMs on MAOs and ChEs were tested. All three MSMs at 10 µM inhibited MAO-A and MAO-B by 35% to 90% inhibition, respectively, with stronger inhibition observed for MAO-B, and with inhibition comparable to the reference compounds selegiline and rasagiline (Figure 1I,J). MTP150 showed the highest inhibition, with an IC_50_ value of 1.50 µM (Figure 1K). Similar results were obtained with AChE and BChE, with preferential inhibition of BChE, and MTP150 achieving 60% inhibition and an IC_50_ of 8.09 µM (Figure 1L–N). Further kinetic analysis of MTP150’s effect on BChE revealed a mixed inhibition mechanism, as indicated by the intersection of the Lineweaver–Burk plot lines in the first quadrant (Figure S1A). Based on the secondary plot, MTP150 presented a Ki of 2.51 µM (Figure S1B). Taken together, these results show that the three MSMs inhibit HDACs, MAOs and ChEs with higher activity against MAO-B and BChE, and highlight MTP150 as the most selective compound.

### 2.2. MTP142, MTP156 and MTP150 MSMs Reduce Cell Proliferation and Promote Apoptosis

**Figure 2:**
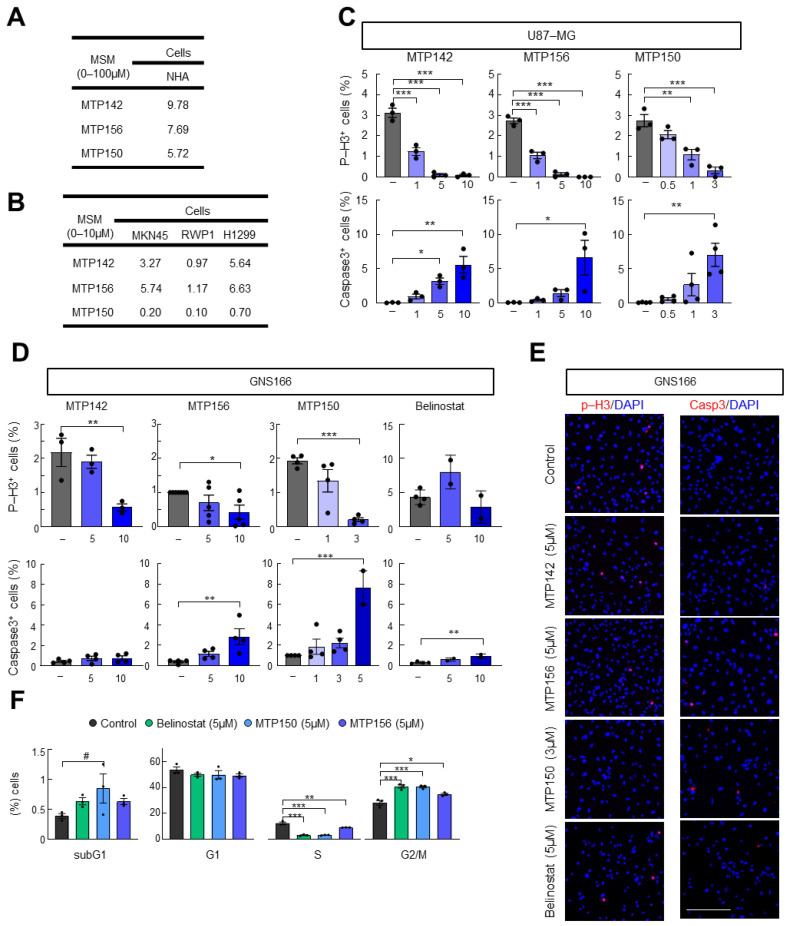
**Novel MTP compounds present antitumor activity.** (**A**) IC50 values of MTP142, MTP156 and MTP150 in NHA. (**B**) IC50 values of MTP142, MTP156 and MTP150 in MKN45, RWP1 and H1299 cell lines. (**C**) Quantification of phospho-Histone 3 and cleaved Caspase 3 marker after the administration of increasing concentrations of MTP142, MTP156 and MTP150 in the U87 cell line (**D**) Quantification of phospho-Histone 3 and cleaved Caspase 3 marker after the administration of increasing concentrations of MTP142, MTP156 and MTP150 in GNS166 cells. (**E**) Representative images of p-H3 and Casp3 markers in GNS166 cells. Scale bar of 200 μm. (**F**) Flow cytometry analysis of GNS166 treated with 5 μM MTP156, MTP150 or Belinostat. # *p* ≤ 0.1, * *p* ≤ 0.05; ** *p* ≤ 0.01; *** *p* ≤ 0.001.

Next, we analyzed the effect of the three MSMs on normal human astrocytes. For this purpose, NHA cells were cultured with increasing concentrations (0–100 μM) of MTP142, MTP156 and MTP150 for 72 h, and IC_50_ values were calculated. We found IC_50_ values for NHAs ranging from 6 to 10 µM, while those for U87 cells ranged from 0.5 to 3 µM (Figure 2A). Additional studies performed in gastric (MKN45), pancreatic (RWP1) and lung (H1299) cancer cells showed that the MSMs were cytotoxic across these models, with IC_50_ values ranging from 0.1 to 7 µM (Figure 2B), supporting the suggestion that the cytotoxic effect of the novel MSMs extends across multiple cancer types. As observed in glioma cells, the lowest IC_50_ values were obtained for MTP150, with values ranging from 0.1 to 0.7 µM (Figure 2B).

We then functionally characterized the effects of MSMs on cell proliferation and apoptosis. To this end, we measured phospho-Histone 3 (p-H3) and cleaved Caspase 3 (Casp3) staining in U87-MG and GNS166 cells. In the proliferation assay, a marked and dose-dependent decrease in p-H3-positive cells was observed with increasing concentrations of all three MSMs (Figure 2C–E and Figure S2A,B). The impairment in cell proliferation was accompanied by a dose-dependent increase in Caspase 3-positive cells in both glioma cells (Figure 2C–E). The comparison with the reference HDAC pan-inhibitor Belinostat revealed a higher effect of the novel MSMs on both impairment of proliferation and enhancement in apoptosis (Figure 2C–E). Flow cytometry was performed to further assess cell cycle alterations following MTP150, MTP156 and Belinostat administration. This analysis revealed an accumulation of cells in the subG0 phase, usually correlated with apoptotic DNA fragmentation, along with a pronounced reduction in cells on the S phase and arrest in the G2/M phase (Figure 2F). These effects were also exacerbated in MTPs compared with Belinostat (Figure 2F).

Since MTP150 was the most selective target inhibitor and the most cytotoxic to tumor cells, we focused our attention on this compound and carried out additional experiments. First, the above-described experiments were extended to additional glioma and GSCs (U251-MG and GNS179 cells). Specifically, MTP150 exerted a potent cytotoxic effect in both cell types, with IC_50_ values of 1 and 6 µM, respectively (Figure S2C). Proliferation and apoptosis analyses mirrored the results obtained in previously tested glioma cells. Thus, proliferation, measured as p-H3- and Ki67-positive cells, was reduced, whereas apoptosis, measured by Caspase 3-positive cells, was increased, both in a dose-dependent manner (Figure S2D,H). Moreover, the comparison of Ki67-positive cells with Belinostat treatment also showed a higher impairment after MTP150 treatment (Figure S2H).

### 2.3. MTP150 Alters Cell Cycle, DNA Remodeling and Synapse Pathways in GSCs

**Figure 3:**
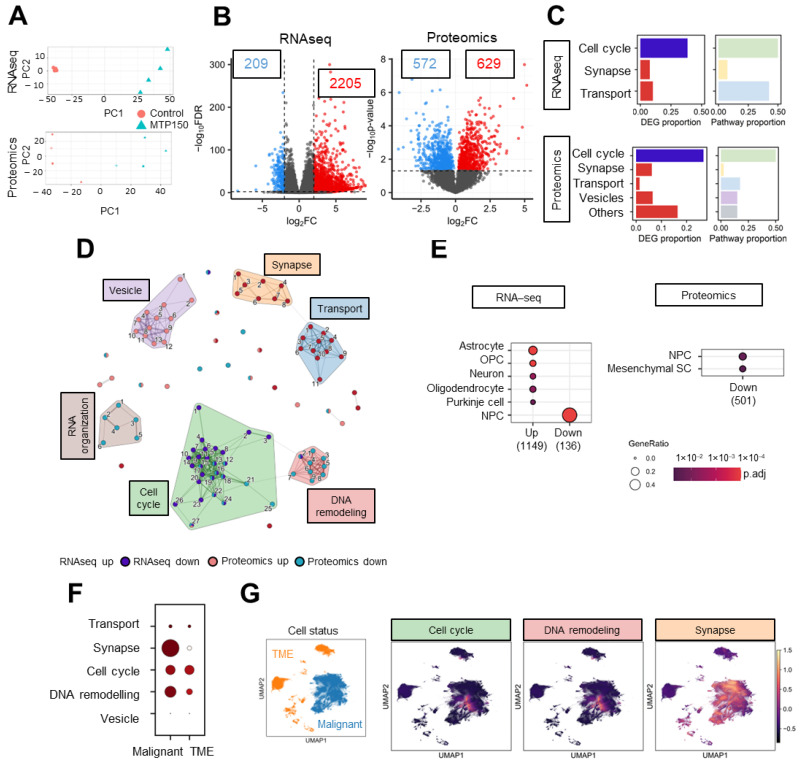
**MTP150 impairs cell cycle, DNA remodeling and synapse in GSCs**. (**A**) PCA plot of RNAseq and proteomics studies. (**B**) Volcano plot of RNAseq and proteomics using a fold-change > 4 and *p*-value < 0.01 for RNAseq, and *p*-value < 0.05 for proteomics. (**C**) Proportion of DEGs and biological processes represented by the biological themes in RNAseq and proteomic studies, respectively. (**D**) Enrichment map representing the connection between pathways associated with DEGs and DEPs in RNAseq and proteomic studies, respectively. The width of the connecting bars indicates the number of genes that are shared between the two pathways. (**E**) Human cell type marker ORA from RNA-seq and proteomic studies, based on data from the CellMarker database. Gene ratio goes from 0.0 to 0.4 and the *p* adjust until 1 × 10^−2^. (**F**,**G**) Uniform Manifold Approximation and Projection (UMAP) visualization displaying the predefined cellular states from the original study, segmented into malignant cells and the tumor microenvironment (TME), and UMAP visualization of the GBMspace dataset depicting the mean module scores derived from the genes commonly differentially expressed (DEGs) within the cell cycle cluster defined by the MTP150 multi-omic analyses.

In order to identify the genes and molecular mechanisms underlying MTP150 activity in GSCs, we conducted RNAseq and proteomic profiling. GNS166 cells were either untreated or treated with 5 µM of MTP150, and both OMIC approaches were analyzed. PCA demonstrated robust segregation between treated and untreated samples (Figure 3A). Using a fold-change cutoff > 4 and *p*-value < 0.01, RNAseq analysis identified a total of 2205 upregulated and 209 downregulated genes in the treated condition. In parallel, proteomics using a *p*-value < 0.05 revealed 572 upregulated and 629 downregulated proteins (Figure 3B). Gene Ontology enrichment analyses were performed separately for RNAseq- and proteomics-derived differentially expressed genes and proteins. In RNAseq, the top 20 enriched biological processes among downregulated genes were strongly associated with cell cycle progression, while upregulated genes were enriched in pathways related to synapse and ion-channel transport and activity (Figure 3C and Figure S3A). Proteomic findings showed a slightly broader distribution of biological processes with pathways associated with cell cycle and DNA organization downregulated, whereas synapse-related processes were upregulated (Figure 3C and Figure S3B). Thus, a reduction in cell cycle pathways was observed across both OMIC layers, providing support for the in vitro functional assays.

Next, a combined analysis of both approaches was performed, for which the top 25 enriched upregulated and downregulated pathways were selected considering all Gene Ontology domains, and clustered using Jaccard similarity. A total of six different clusters were obtained. Notably, the two largest downregulated clusters were linked to cell cycle regulation and DNA remodeling, while the principal upregulated cluster was related to synapse (Figure 3D). In order to further characterized the molecular mechanism underlying MTP150 treatment, we moved to human glioblastoma sc-RNAseq public available datasets. Using the CellMarker database, we identified that genes and proteins decreased after MTP150 treatment were decreased in clusters linked to NPCs (neural progenitor cells) and increased on neurons (Figure 3E). In addition, data from the GBM Space resource (https://www.gbmspace.org/) allowed us to identify that the set of genes/proteins of all clusters, especially cell cycle, DNA remodeling and synapse, were more expressed in malignant cells than in the TME (Figure 3F,G and Figure S4A–C).

**Figure 4:**
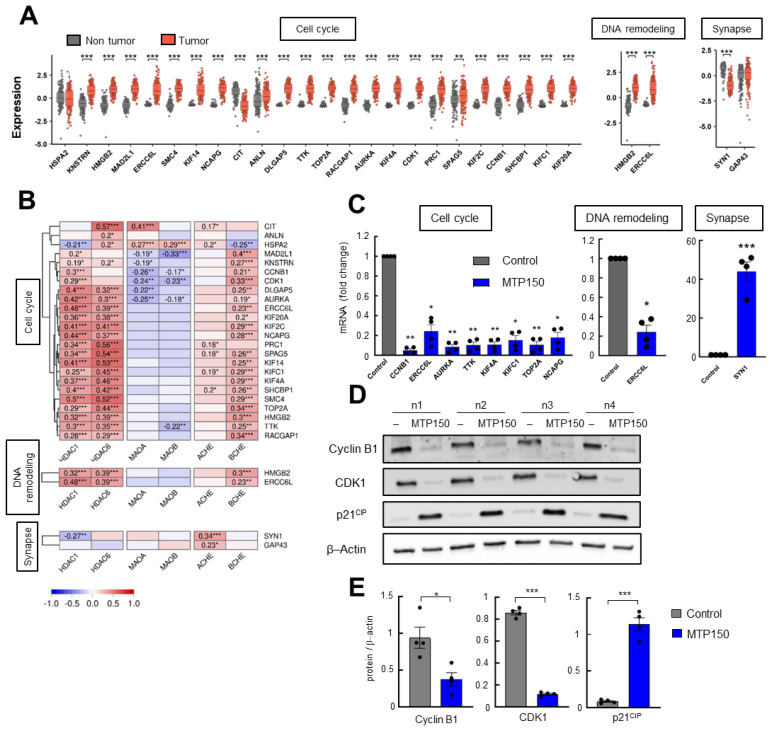
(**A**) Normal versus tumor expression of common DEGs of the Cell cycle, DNA remodeling and Synapse clusters in the TCGA cohort. (**B**) Correlation analysis of DEGs from selected clusters with target genes of MTP150 in samples from TCGA cohort. (**C**) qRT-PCR of selected DEGs from each cluster in GNS166 non-treated and MTP150 treated cells (*n* = 4). (**D**,**E**) Western Blot analysis and their quantification of indicated proteins involved in cell cycle (*n* = 4). * *p* < 0.05; ** *p* < 0.01; *** *p* < 0.001.

Next, we validated the differentially expressed genes shared by both OMICs. First, the levels of those exhibiting altered expression in both datasets were examined in TCGA human glioblastoma samples. All genes, but one, from the cell cycle and DNA remodeling clusters showed elevated expression levels in glioblastoma compared with control tissue (Figure 4A). Additionally, correlation analyses with HDACs, MAOs, and ChEs MSMs targets revealed that genes associated with cell cycle and DNA remodeling showed significant positive correlations mainly with HDACs, as well as with BChE (Figure 4B). Next, the expression of the DEGs was validated by qRT-PCR in GNS166 cells. Consistent with the OMIC findings, genes related to cell cycle and DNA remodeling were significantly downregulated, while the expression of the genes associated with synapse were increased in cells treated with MTP150 (Figure 4C). Finally, relevant selected DEPs linked to cell cycle such as Cyclin B1, CDK1, KI67 and p21^CIP^ were studied by WB, confirming their deregulation in response to MTP150 in GNS cells (Figure 4D,E and Figure S5).

### 2.4. MTP150 Reduces Tumor Growth In Vivo Alone or in Combination with TMZ

**Figure 5:**
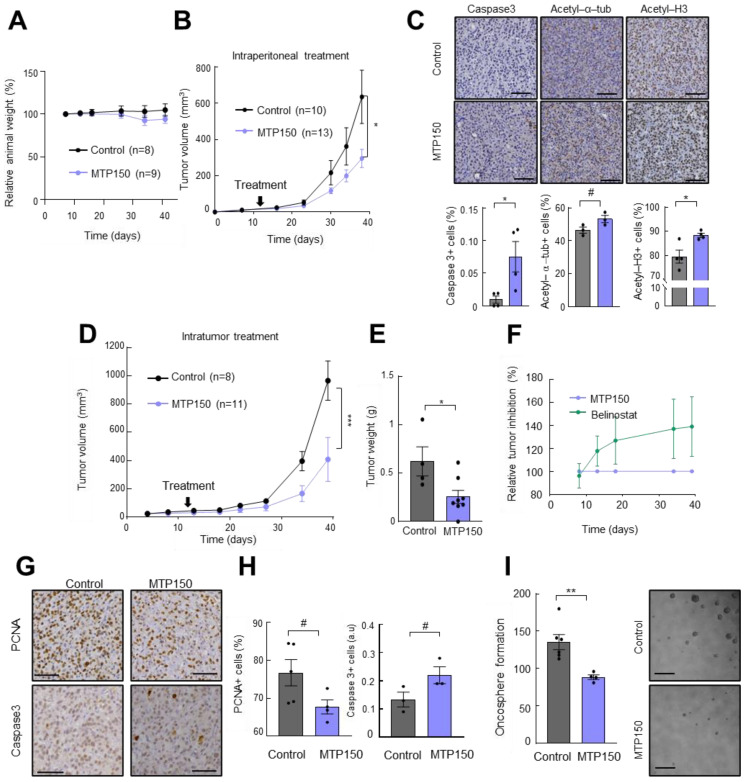
**MTP150 reduces tumor growth in vivo.** (**A**) Relative change in body weight of mice treated with vehicle or 15 mg/Kg of MTP150 (*n* ≥ 8). (**B**) Measurement of tumor volume at indicated timepoints of mice intraperitoneally treated with vehicle or 200 µM of MTP150 (*n* ≥ 10). (**C**) Representative immunohistochemistry and quantification of Ki67, cleaved Caspase 3, acetyl-a-Tub and acetyl H3 at the end-point (*n* ≥ 3). Scale bar represents 200 µm. (**D**) Measurement of tumor volume at the indicated timepoints of mice intratumorally treated with vehicle or 200 µM of MTP150 (*n* ≥ 8). (**E**) Tumor weight at the end-point day 39 (*n* ≥ 4). (**F**) Relative tumor inhibition of animals treated with MTP150 or Belinostat. (**G**,**H**) Representative immunohistochemistry and quantification of PCNA and cleaved Caspase 3 at the end-point (*n* ≥ 3). Scale bar represents 200 µm. (**I**) Quantification of oncospheres derived from tumors of controls and MTP150 treated animals (*n* ≥ 4). Scale bar represents 100 µm. # *p* ≤ 0.1, * *p* ≤ 0.05; ** *p* ≤ 0.01; *** *p* ≤ 0.001.

Next, we moved to in vivo studies and the cytotoxicity of MTP150 was assessed by intraperitoneal injection of 15 mg/mL of MTP150 over 4 weeks. The treatment did not produce any significant changes in animal body weight compared to controls throughout the experiment (Figure 5A), indicating good systemic tolerability. In line, macroscopic analysis did not show clear differences in post-mortem tissues. Consequently, the antitumor effect of MTP150 was next assessed in vivo initially by single-treatment experiments. Briefly, U87-MG cells were subcutaneously injected in immunodeficient mice, and once tumors were formed, mice were grouped and treated intraperitoneally. MTP150 significantly inhibited tumor growth in this assay, with treated tumors reaching an average volume of ~270 mm^3^ compared to ~650 mm^3^ in the control group (Figure 5B). To reinforce these results, we performed immunohistochemical analysis on extracted tumors. The staining of acetyl-α-tubulin and acetyl H3 showed that MTP150 promotes an increase in both in the tumors (Figure 5C), demonstrating the in vivo targeting of the MSM. Moreover, cleaved Caspase 3 staining showed an increase in apoptosis on MTP150-treated animals (Figure 5C). Afterwards, another experiment was performed in which tumors were treated intratumorally with vehicle or 200 µM of MTP150, once stablished, allowing for direct assessment of local drug efficacy. Notably, MTP150 significantly reduced tumor growth, resulting in an average volume of ~480 mm^3^ compared to ~900 mm^3^ in the control group. This represents a ~50% reduction in final tumor volume (Figure 5D). In line, this effect was also reflected in the tumor weight, with MTP150-treated tumors showing >40% lower mass than controls (Figure 5E). Moreover, the comparison with the tumor growth promoted by Belinostat treatment revealed a more intense tumor inhibition of MTP150 compared with Belinostat (Figure 5F). Immunohistochemical staining of resected tumors showed that MTP150-treated tumors displayed fewer PCNA positive cells and increased cleaved Caspase 3 compared to controls (Figure 5G,H), showing the impaired proliferation and enhanced apoptosis in vivo. Moreover, cells dissociated from MTP150-treated tumors generated significantly fewer oncospheres than controls after 10 days in the absence of treatment (Figure 5I). Together, these results show the sustained antitumor activity of the novel MSM compound.

**Figure 6:**
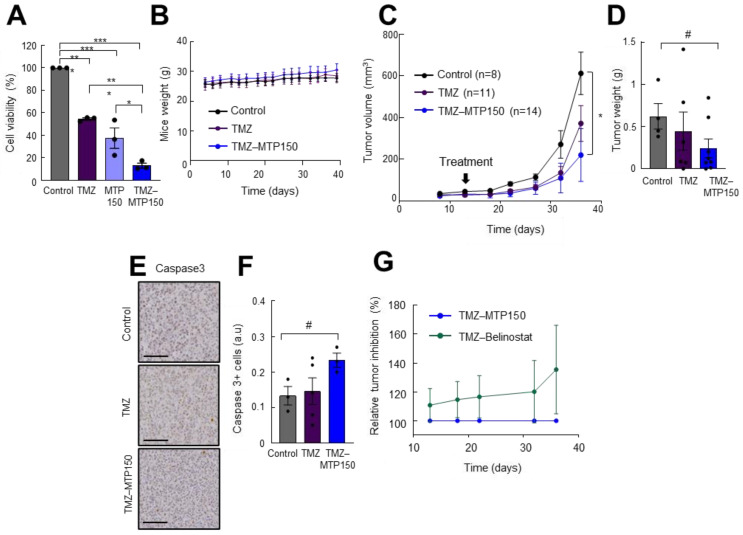
**MTP150 plus TMZ reduces tumor growth in vivo** (**A**) Cell viability assay of cells treated for 72 h with TMZ and MTP150, alone and in combination (*n* = 3). (**B**) Control, TMZ and MTP150-TMZ treated mice weight along the experiment. (**C**) Measurement of tumor volume at the indicated timepoints of mice treated with vehicle, TMZ or MTP150-TMZ (*n* ≥ 8). (**D**) Tumor weight at the end-point (*n* ≥ 4). (**E**) Representative immunohistochemistry and (**F**) quantification of cleaved Caspase 3 at end-point (*n* ≥ 3). Scale bar 200 μm. (**G**) Relative tumor inhibition of TMZ-MTP150 and TMZ-Belinostat treated animals. # *p* ≤ 0.1, * *p* ≤ 0.05; ** *p* ≤ 0.01; *** *p* ≤ 0.001.

Next, the effect of MTP150 was tested in combination with TMZ. For in vitro experiments, 250 µM TMZ and 0.5 µM MTP150 were used and the results revealed that both compounds alone significantly reduced cell viability compared to untreated cells. However, the reduction was significantly enhanced when both agents were combined. Cell viability decreased to 15%, compared with 55% and 37% for TMZ and MTP150 alone, respectively, as measured by the MTT assay (Figure 6A). We then moved to the in vivo setting and combined MTP150 with TMZ. We evaluated the weight of animals, showing no toxicity effect in mice due to the low dose of the compounds (Figure 6B). As expected, and consistent with previous reports, TMZ alone reduced tumor volume compared to controls (Figure 6C). Noteworthy, the antitumor effect was further increased when combined with MTP150. Tumors treated with TMZ alone had an average volume of ~580 mm^3^, while the TMZ + MTP150 combination reduced tumor volume to ~445 mm^3^, corresponding to reductions of ~50% versus control and ~25% versus TMZ alone, respectively (Figure 6C). In addition, tumor weights from the combined treatment group were nearly 50% lower than controls (Figure 6D). Immunohistochemical staining revealed that tumors treated with the combination of TMZ + MTP150 displayed increased cleaved Caspase 3-positive cells compared to other groups (Figure 6E,F). Finally, the comparison of MTP150 with Belinostat treatment demonstrated that MTP150 exerted greater antitumor activity both as monotherapy and in combination with TMZ (Figure 6G). These results reveal the potent antitumor activity of MTP150 MSM alone and in combination with TMZ.

## 3. Discussion

Complex diseases such as cancer are characterized by diverse molecular alterations, which limit the effectiveness of single-target treatments [10]. The marked intratumoral heterogeneity, highly infiltrative behavior, and the presence of GSCs also contribute to the poor patient prognosis. The development of more effective and mechanistically rational therapeutic strategies remains an unmet clinical need [37,38]. In this study, we characterize the antitumor activity of a novel family of pentatarget MSMs in cancer, with a particular focus on glioblastoma. These compounds integrate Contilisant’s ability to inhibit MAOs and ChEs and to modulate H_3_R and S1R [17] with Belinostat’s pan-HDAC inhibitory activity [31]. These targets have been reported to be consistently deregulated in glioblastoma, and thus, they are targets for the treatment of the disease [27,39,40,41,42]. Among the nine members studied, three MSMs were prioritized, with particular emphasis on MTP150 due to its superior target selectivity and pronounced cytotoxic effect, observed not only in proliferating glioma cells but also in GSCs, the subpopulation responsible for therapeutic resistance and recurrence [43].

In particular, MTP150 presented greater inhibitory potency against HDACs than Belinostat and SAHA, and exerted higher inhibitory effects on MAO-B and BChE compared to the other MAOs and ChEs examined. MAO-B is considered a key target in glioma, and studies on MAO-B-activated prodrugs have shown therapeutic benefit in glioblastoma models [44,45]. Regarding ChEs, inhibitors of these enzymes have been proposed to provide additional advantages for patients with glioblastoma, as they can enhance cognitive functions, thereby mitigating the neurotoxic effects frequently associated with glioblastoma treatment [46]. Although the effect of the MSMs on H_3_R and S1R has not yet been fully characterized, prior research indicates that both receptors are deregulated in glioblastoma and suggests that their modulation may hold therapeutic relevance [47,48]. Thus, some of the biological effects observed in this work may be partially attributable to MSM activity on H_3_R and S1R receptors. In this regard, there are several studies on multitarget molecules that combine the modulation of some of the enzymes and receptors targeted by our MSMs for glioblastoma. For example, a dual MAO-A and HDAC inhibitor reduced cell growth and induced cell death in glioma cells, prolonging survival in vivo [29]. Dual modulators combining S1R agonism with HDAC inhibition have also exerted antitumor activity in glioblastoma, inducing cell death in both TMZ-sensitive and -resistant cells [30]. Along these lines, we recently generated and described a novel molecule combining Contilisant and SAHA, which displayed significant antitumor activity in GSCs both in vitro and in vivo [42].

Functionally, the three selected MSMs, particularly MTP150, reduced proliferation and induced apoptosis in a dose-dependent manner in both GB cells and GSCs. These phenotypic effects were supported by OMICs analyses, which showed that MSM treatment deregulated gene networks governing the cell cycle and DNA remodeling. The effects on the cell cycle are a major characteristic of HDAC inhibition, and previous studies have shown that HDAC inhibitors modulate the expression of key cell cycle regulators such as cyclin B1, CDKs, INK4a/ARF locus, p21^CIP^, Ki67, and Aurora kinase [23,42,49]. Moreover, MTP150 promoted a significant reduction in tumor volume in vivo, both when administered alone and in combination with TMZ, while exerting no apparent toxic effects on the animals. However, the validation of its antitumor efficacy was not tested in orthotopic models nor was an experimental test of the BBB crossing carried out. Moreover, no detailed histopathological analysis of organs was completed to further confirm the toxicity data. These results align with reports showing that Belinostat, the HDAC inhibitor used for the synthesis of MTP150, was effective in reducing tumor growth in an orthotopic glioma rat model [50]. Similarly, MSMs generated by the juxtaposition of Contilisant and Tubastatin A or SAHA have been shown to act on their expected targets and either improve cognition or suppress tumor progression [35,42]. Notably, the antitumor effects of MTP150 on proliferation and tumor growth, both in vitro and in vivo, surpassed those of Belinostat alone, supporting the therapeutic advantage of the MSM approach.

To sum up, this work describes a novel family of MSMs designed to simultaneously inhibit HDACs, MAOs, ChEs, H_3_R, and S1R by combining Contilisant and Belinostat into a single multitarget scaffold. Among them, MTP142, MTP156, and especially MTP150 demonstrated potent cytotoxic activity against proliferating glioblastoma cells and GSCs, reducing cell proliferation and inducing apoptosis. In vivo studies further demonstrated that MTP150 reduced tumor volume both as a monotherapy and in combination with TMZ. In conclusion, our data reveal the preclinical efficacy of a new class of multitarget small molecules against glioblastoma.

## 4. Materials and Methods

### 4.1. Cell Lines and Cultures

U87-MG, U251-MG and NCI-H1299 cells were purchased from ATCC (Manassas, VA, USA). RWP1 cells were kindly provided by Dr. Francisco Real. MKN45 was obtained from the Leibniz Institute DSMZ-German Collection of Microorganism and Cell Cultured (Braunschweig, Germany). All cell lines were cultured in DMEM or RPMI (Gibco, Waltham, MA, USA), supplemented with 10% FBS, 100 U/mL penicillin and streptomycin, and 2 mM L-Glutamine. Patient-derived GSCs (GNS166 and GNS179), kindly provided by Dr. Steve Pollard (MRC Centre for Regenerative Medicine and Edinburgh Cancer Research, Edinburgh, UK) and described elsewhere [51], were cultured in adhesion in DMEM/F12 media (Gibco) supplemented with N2 (Gibco), B27 (Gibco), D-(+)-Glucose solution 45% (Sigma-Aldrich, St. Louis, MO, USA), basic Fibroblast Growth Factor (Gibco), and epidermal growth factor (Sigma-Aldrich). Normal Human Astrocytes (NHAs) were purchased from ScienCell (Carlsbad, CA, USA) and cultured using astrocyte medium kit (ScienCell) supplemented with FBS and Astrocyte Growth Supplement (AGS). All cultures were maintained at standard conditions of 37 °C, 95% humidity, 21% O_2_ and 5% CO_2_, and were regularly tested for *Mycoplasma*.

### 4.2. Compounds

Novel MSMs characterized in this work were synthesized as previously reported [31]. Belinostat and SAHA (Cayman, Ann Arbor, MI, USA) were used as reference HDAC inhibitors and TMZ (Sigma-Aldrich) was employed for combined in vitro and in vivo experiments. All compounds were dissolved in DMSO and stored in aliquots at −20 °C.

### 4.3. Western Blot

Sodium dodecyl sulfate-polyacrylamide gel electrophoresis was performed. Primary antibodies against HDAC1 (Abcam, Waltham, MA, USA) and HDAC6 (Cell Signaling, Danvers, MA, USA), along with their downstream targets, acetylated H3 (Cell Signaling) and acetylated α-tubulin (Abcam), were used. β-actin (Sigma-Aldrich) served as endogenous control. Secondary antibodies conjugated to horseradish peroxidase (anti-rabbit or anti-mouse; Cell Signaling) were applied. Proteins were detected using the iBright imaging system and either NOVEX ECL Chemi Substrate or SuperSignal West Femto Maximum Sensitivity Substrate (ThermoFisher, Waltham, MA, USA).

### 4.4. Study of Cell Viability

Cells were seeded at a density of 1.5 × 10^3^ cells/well (conventional cell lines) or 5 × 10^3^ cells/well (GNS and NHA) in 96-well plates and incubated overnight. Cells were then treated with increasing concentrations of MSMs. After 72 h, cells were incubated for 3.5 h with MTT, after which absorbance at 570 nm was measured. IC_50_ values were calculated using GraphPad Prism software.

### 4.5. Enzyme Inhibition Assays

For MAO inhibition, an inhibitor solution containing 10 µM of the MSMs was incubated with the enzyme solution (0.5 U/mL recombinant hMAO-A or 1.5 U/mL recombinant hMAO-B) for 30 min at 37 °C. Subsequently, 200 U/mL horseradish peroxidase, 20 mM Ampliflu Red and 100 mM tyramine hydrochloride (Sigma-Aldrich) were added, and absorbance was measured at 570 nm every 5 min for 30 min. The inhibition percentage was calculated from these absorbance values. For compounds exhibiting >50% inhibition, IC_50_ values were determined by repeating the protocol with increasing inhibitor concentrations.

To assess ChEs inhibition, Ellman’s colorimetric method was followed. Briefly, an inhibitor solution containing 10 µM MSMs and 0.3 mM DTNB was incubated for 20 min with an enzyme solution (0.5 U/mL recombinant hMAO-A or 0.25 U/mL recombinant hMAO-B) at 37 °C. After incubation, the substrate solution (acetylthiocholine or butyrylthiocholine iodide, Sigma-Aldrich, at 1.5 mM) was added, and absorbance was recorded at 405 nm every minute for 10 min. The percentage of inhibition was calculated, and IC_50_ values were determined for compounds showing >50% inhibition using increasing compound concentrations. Enzyme kinetics were evaluated with varying substrate and inhibitor concentrations, and the results were plotted as Lineweaver–Burk plots using GraphPad Prism software. The inhibition constant (K_i_) was estimated from the intersection on the *x*-axis in secondary plots.

### 4.6. Immunofluorescence

For immunofluorescence assays, cells were seeded at a density of 2 × 10^4^ cells/well in 8-well immunofluorescence chambers (LabTek Thermo, Waltham, MA, USA) and incubated overnight. Following 48 h of treatment, cells were fixed with 4% PFA. Primary antibodies against phospho-Histone H3 (p-H3, Abcam) and Caspase 3 (R&D Systems, Minneapolis, MN, USA) and secondary antibodies Alexa Fluor anti-mouse and anti-rabbit (Invitrogen, Carlsbad, CA, USA) were used. Nuclei were stained with DAPI (Sigma-Aldrich). Images were acquired using an Axio Observer 7 epifluorescence microscope, and quantification was performed with Qupath software version 0.6.0.

### 4.7. Cell Cycle Analysis by Flow Cytometry

Cells were harvested using accutase and washed with PBS. Then, 1·10^6^ cells were fixed with 70% cold ethanol at −20 °C for at least 24 h, and incubated with 0.5% Triton X-100 and 25 µg/mL RNase A in PBS for 30 minutes at room temperature. Then, DNA was stained with 25 ng/mL propidium iodide for 15 minutes and samples were analyzed using a CytoFLEX flow cytometer (Beckman Coulter Co., Brea, CA, USA).

### 4.8. Online Prediction Tools

Physicochemical properties of the compounds of study, as well as Lipinski rule violations, were predicted by entering structures in Molinspiration (https://www.molinspiration.com/cgi/properties, accessed on 15 November 2024). The ability to cross the BBB was predicted using Online BBB Predictor (http://ssbio.cau.ac.kr/software/logbb_pred/, accessed on 15 November 2024) with default parameters. Potential toxicity risks were assessed using OSIRIS Property Explorer (https://www.organic-chemistry.org/prog/peo/, accessed on 15 November 2024).

### 4.9. RNAseq and Proteomic Studies

RNAseq and proteomic analyses were performed following treatment with 5 µM MTP150 in GNS166 cells. For transcriptomics, total RNA was extracted and submitted to BGI Tech Solutions. On average, 44 million clean reads were obtained per sample. Differential expression analysis was performed with DESeq2 (version 1.44.0) using default parameters. Genes with a fold-change value ≥ 4 or ≤−4 and FDR-adjusted *p*-value < 0.01 were considered differentially expressed and included in downstream enrichment analysis. The RNAseq dataset was deposited in GEO (GSE283024).

Proteomics was performed using label-free relative quantification on nLC-MS/MS at the Proteomic Facility of CIC bioGUNE. Overrepresentation analysis of significantly differentially expressed proteins (*p* < 0.05) was conducted with *clusterProfiler* version 4.16.0 R package. The proteomic dataset is available via the PRIDE partner repository (PXD058042).

In both RNAseq and proteomics, the top 20 enriched terms were visualized. Combined analyses were conducted, including enrichment analyses for up- and downregulated genes/proteins (all Gene Ontology domains, all genes as background). The top 25 enriched gene sets were clustered by Jaccard distances. Genes/proteins significantly differentially expressed in both datasets for each cluster were identified, and Spearman’s correlation analyses were performed between these and compound targets using TCGA data.

### 4.10. RNA Extraction and RT-qPCR

Total RNA was extracted using Trizol (Life Technologies, Carlsbad, CA, USA). Reverse transcription (RT) was performed using the Maxima First Strand cDNA Synthesis Kit (ThermoFisher), following the manufacturer’s guidelines. Quantitative PCR (qPCR) was carried out in a CFX384 Thermal Cycler (BioRad, Hercules, CA, USA) using Absolute SYBR Green mix (ThermoFisher). 18S served as housekeeping gene for normalization, and the 2^−ΔΔCt^ method was used for relative quantification.

### 4.11. In Vivo Toxicity Assay

Briefly, 15 mg/kg of MTP150 or vehicle (DMSO) was administered intraperitoneally to athymic nude-Foxn1^nu^ mice on a 5-days-on, 2-days-off schedule. Body weight was monitored to assess MSM potential toxicity. At the end-point, macroscopic analysis of the different organs was carried out.

### 4.12. In Vivo Carcinogenesis Assays

Subcutaneous tumor growth was initiated by injecting 5 × 10^5^ cells into both hind flanks of athymic nude-Foxn1^nu^ mice. Once the tumors reached 25–50 mm^3^, mice were allocated to treatment groups by gender and tumor size. For monotherapy, mice were treated intratumorally with vehicle (DMSO dilution) or 200 µM of MTP150 or intraperitoneally with 15 mg/kg, on a 5-days-on, 2-days-off schedule. In combination therapy, 0.5 mg/Kg TMZ or vehicle was administered intraperitoneally during the first 5 days; intratumoral MTP150 or vehicle was delivered with the same schedule. After 4 weeks, mice were sacrificed and tumors were extracted.

### 4.13. Statistical Analysis

GraphPad Prism software (Version 8.3.0, San Diego, CA, USA) was used for all statistical analyses. Data are presented as mean ± standard error of the mean (SEM). Unless noted otherwise, differences between ≥3 groups were analyzed by one-way ANOVA with Dunnett’s post hoc test (comparison to control). In vivo tumor growth was evaluated by two-way ANOVA using a mixed-effect model. Significance was defined as *p* < 0.05 (#, *p* ≤ 0.1; * *p* ≤ 0.05; ** *p* ≤ 0.01; *** *p* ≤ 0.001).

## Data Availability

The original contributions presented in this study are included in the article/Supplementary Materials. Further inquiries can be directed to the corresponding author.

## Supplementary Materials

The following supporting information can be downloaded at: https://www.mdpi.com/article/10.3390/ph19010020/s1, Figure S1: (A) Lineweaver-Burk plot representing reciprocal of velocity versus reciprocal of substrate concentrations at different concentrations of MTP150. (B) Secondary plot of slopes obtained in Lineweaver-Burk plot versus different inhibitor concentrations for the estimation of Ki for MTP150. Figure S2: (A,B) Representative images of (A) p-H3 and (B) Casp3 markers after MTP142, MTP156 and MTp150 administration in U87 cell line. Scale bar is 200 μm. (C) IC50 of MTP150 in U251 and GNS179 cell lines. (D,E) quantification of p-H3 or Casp3 positive cells after MTP150 or Belinostat administration in GNS179 cells, (F,G) quantification of p-H3 and Casp3 positive cells after MTP150 administration in U251 cell line. (H) Quantification of Ki67 positive cell after MTP150 or Belinostat administration in GNS166 and GNS179 cell lines. Figure S3: (A,B) Gene ontology study of RNAseq and proteomics representing the 20 most correlated pathways with upregulated and downregulated DEGs. Figure S4: (A) Uniform Manifold Approximation and Projection (UMAP) visualization of the different cell type defined in the publicly available dataset. (B) UMAP visualization of Transport and Vesicle clusters defined by the MTP150 multi-omic analysis. (C) UAMP associated heatmap depicting the mean module scores derived from the genes commonly differentially expressed (DEGs). Figure S5: (A) Expression at RNA and protein levels of CDKN1A and MKI67 in the RNA-seq and proteomic studies. (B) WB membrane of Ki67 protein and quantification between GNS166 treated with MTP150 and control samples.
