# Supplementary material for: Characterization of a Novel Family of Contilisant + Belinostat Multitarget Small Molecules in Glioblastoma
Source: Pharmaceuticals (Basel). 2025 Dec 22;19(1):20. doi: 10.3390/ph19010020 (PMC12845422; doi:10.3390/ph19010020)
Supplement: Supplementary file 1 [file pharmaceuticals-19-00020-s001.zip › pharmaceuticals-3997006-supplementary.pdf]

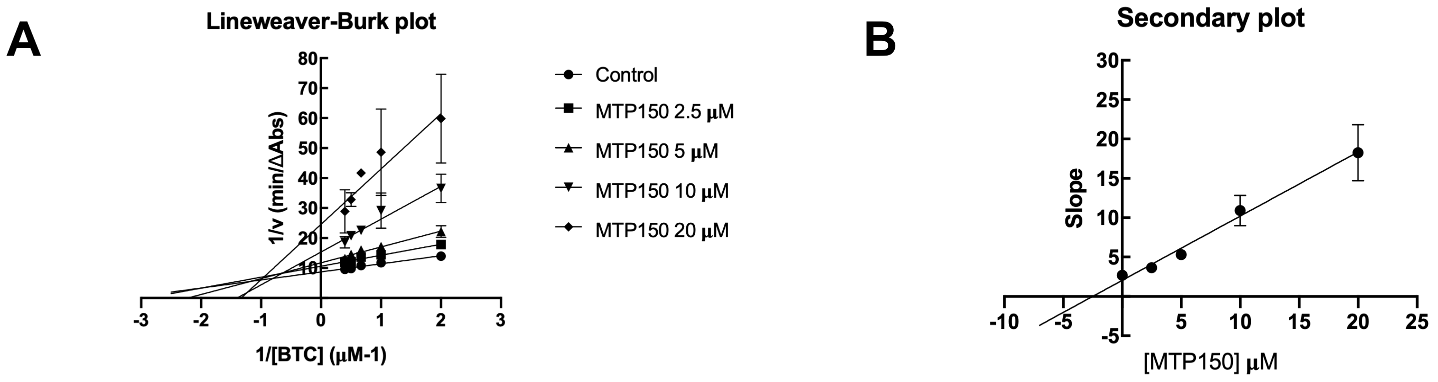

Figure S1. A) Lineweaver-Burk plot representing reciprocal of velocity versus reciprocal of substrate concentrations at different concentrations of MTP150. **B)** Secondary plot of slopes obtained in Lineweaver-Burk plot versus different inhibitor concentrations for the estimation of Ki for MTP150.

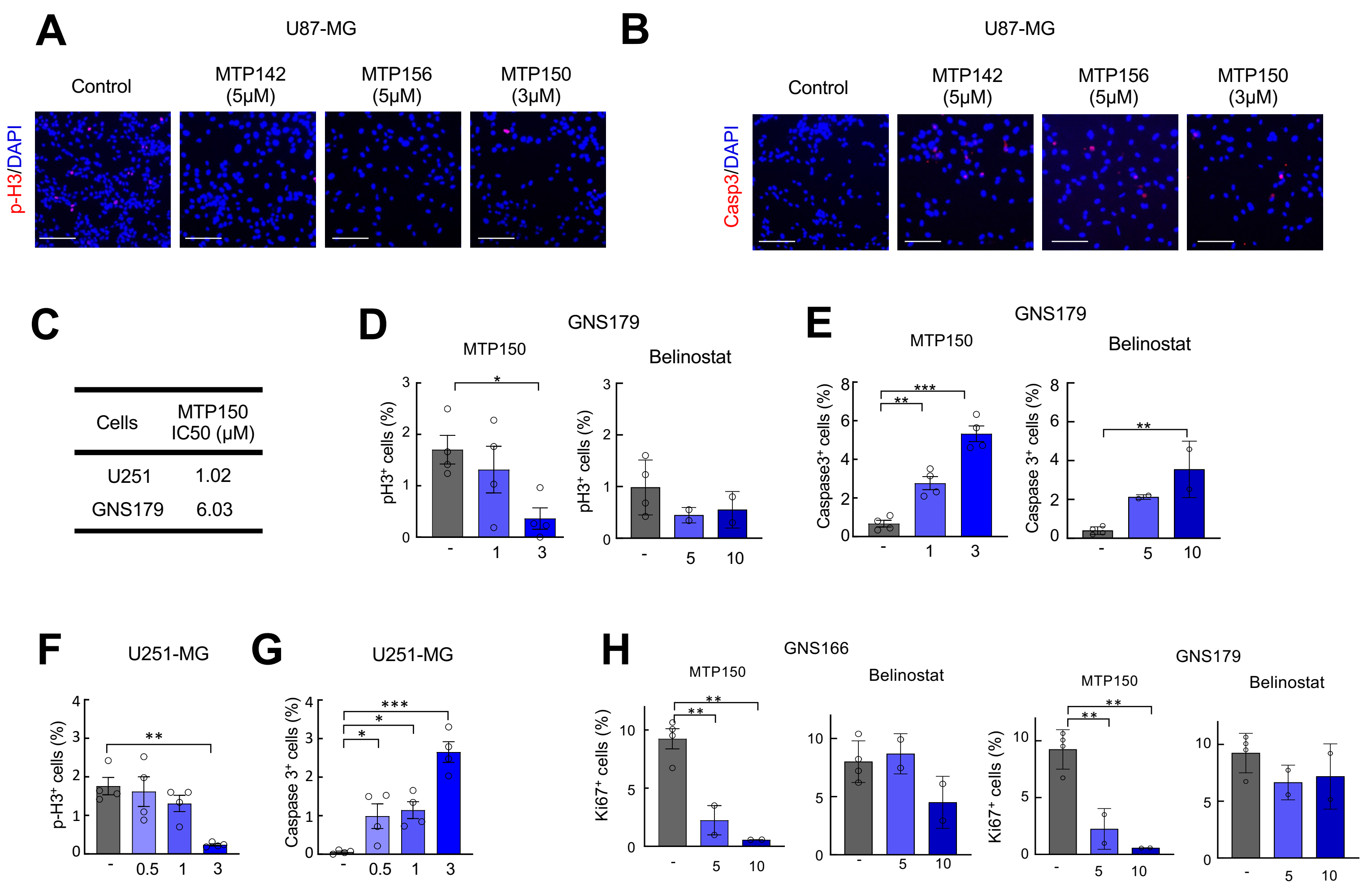

Figure S2. A-B) Representative images of (A) p-H3 and (B) Casp3 markers after MTP142, MTP156 and MTp150 administration in U87 cell line. Scale bar is 200μm. **C)** IC_{50} of MTP150 in U251 and GNS179 cell lines. **D-E)** quantification of p-H3 or Casp3 positive cells after MTP150 or Belinostat administration in GNS179 cells, **F-G)** quantification of p-H3 and Casp3 positive cells after MTP150 administration in U251 cell line. **H)** Quantification of Ki67 positive cell after MTP150 or Belinostat administration in GNS166 and GNS179 cell lines.

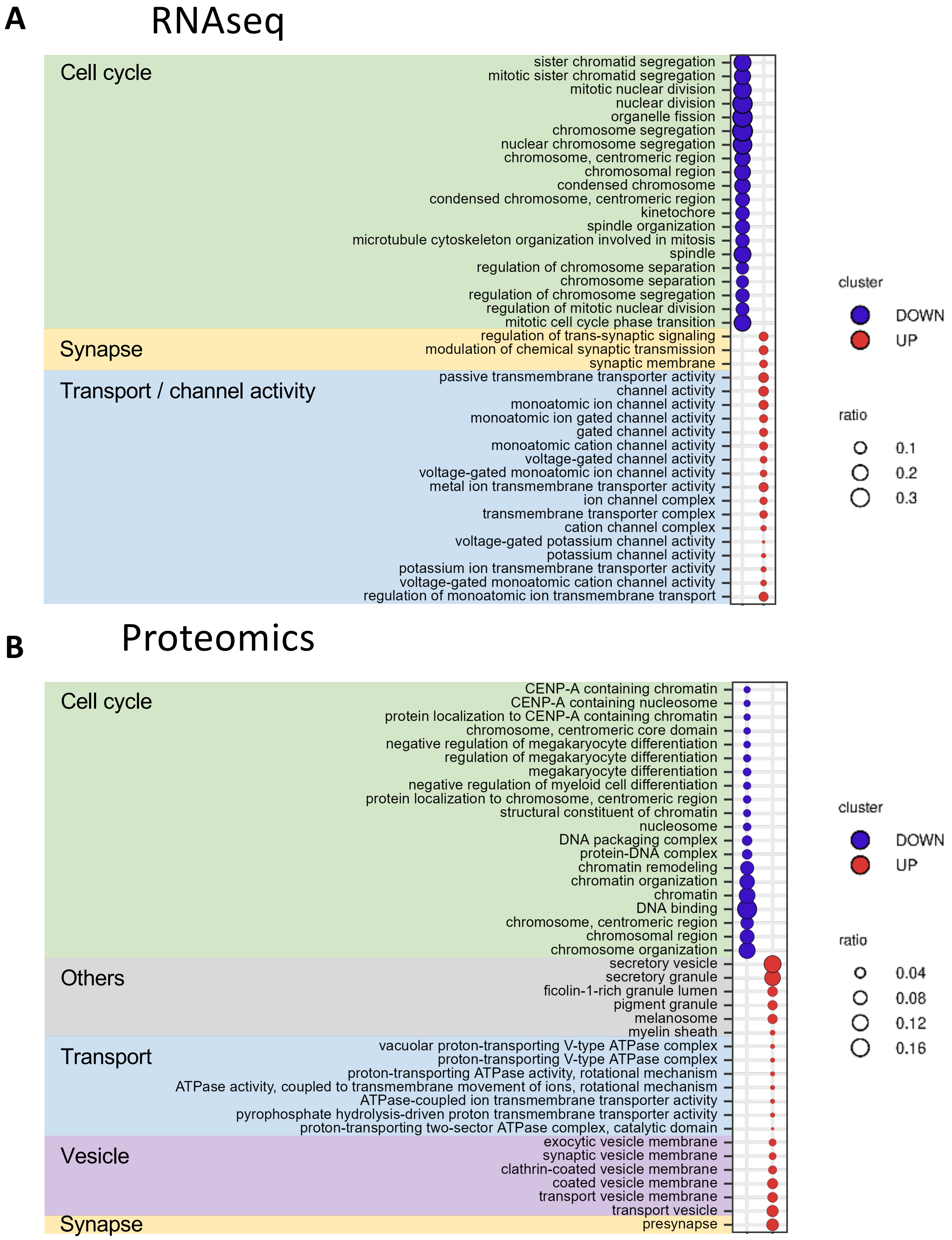

Figure S3. A-B) Gene ontology study of RNAseq and proteomics representing the 20 most correlated pathways with upregulated and downregulated DEGs.

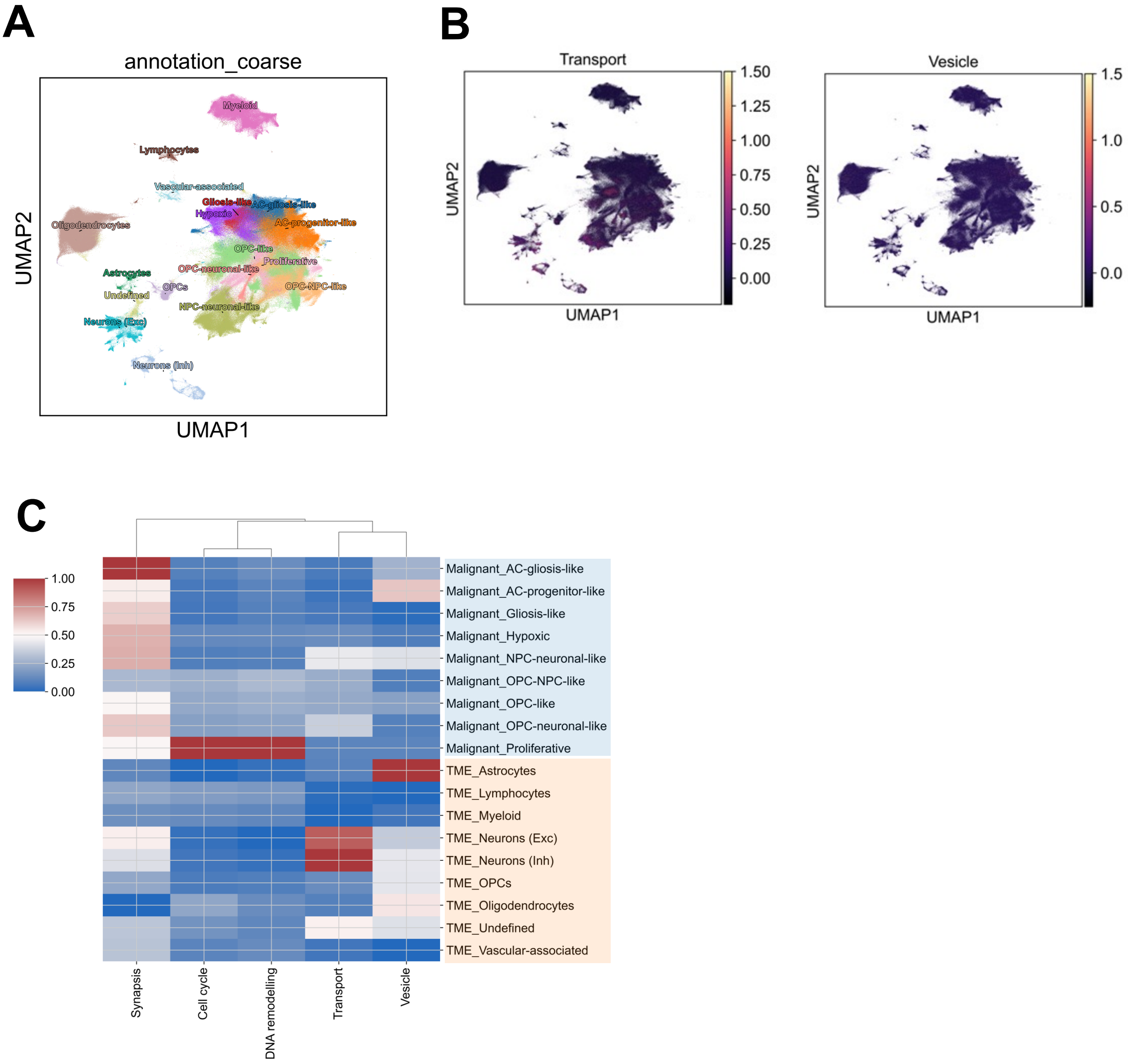

Figure S4. A) Uniform Manifold Approximation and Projection (UMAP) visualization of the different cell type defined in the publicly available dataset. **B)** UMAP visualization of Transport and Vesicle clusters defined by the MTP150 multi-omic analysis. **C)** UAMP associated heatmap depicting the mean module scores derived from the genes commonly differentially expressed (DEGs)

A

Compound	Gene	FC	padj
RNAseq	CDKN1A	2.43	1.68E-71
RNAseq	MKI67	0.16	7.16E-16

Compound	Gene	FC	p-value
Proteomics	CDKN1A	4.45	0.004
Proteomics	MKI67	0.63	0.17

B

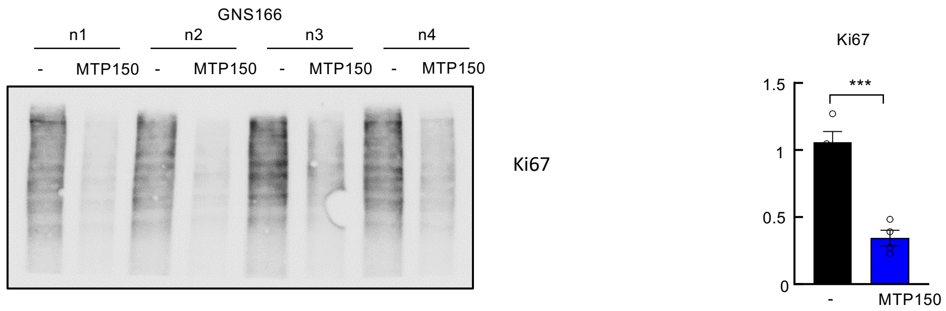

Figure S5. A) Expression at RNA and protein levels of CDKN1A and MKI67 in the RNA-seq and proteomic studies. **B)** WB membrane of Ki67 protein and quantification between GNS166 treated with MTP150 and control samples
